# Genomic prediction of plant traits by popular machine learning methods

**DOI:** 10.18699/vjgb-25-49

**Published:** 2025-06

**Authors:** K.N. Kozlov, M.P. Bankin, E.A. Semenova, M.G. Samsonova

**Affiliations:** Peter the Great St. Petersburg Polytechnic University, St. Petersburg, Russia; Peter the Great St. Petersburg Polytechnic University, St. Petersburg, Russia; Far Eastern State Agrarian University, Blagoveshchensk, Amur region, Russia; Peter the Great St. Petersburg Polytechnic University, St. Petersburg, Russia

**Keywords:** genomic prediction, plant phenotype, machine learning, deep learning, artificial intelligence, геномное прогнозирование, фенотип растений, машинное обучение, глубокое обучение, искусственный интеллект

## Abstract

A rapid growth of the available body of genomic data has made it possible to obtain extensive results in genomic prediction and identification of associations of SNPs with phenotypic traits. In many cases, to identify new relationships between phenotypes and genotypes, it is preferable to use machine learning, deep learning and artificial intelligence, especially explainable artificial intelligence, capable of recognizing complex patterns. 80 sources were manually selected; while there were no restrictions on the release date, the main attention was paid to the originality of the proposed approach for use in genomic prediction. The article considers models for genomic prediction, convolutional neural networks, explainable artificial intelligence and large language models. Attention is paid to Data Augmentation, Transfer Learning, Dimensionality Reduction methods and hybrid methods. Research in the field of model-specific and model-independent methods for interpretation of model solutions is represented by three main categories: sensing, perturbation, and surrogate model. The considered examples reflect the main modern trends in this area of research. The growing role of large language models, including those based on transformers, for genetic code processing, as well as the development of data augmentation methods, are noted. Among hybrid approaches, the prospect of combining machine learning models and models of plant development based on biophysical and biochemical processes is emphasized. Since the methods of machine learning and artificial intelligence are the focus of attention of both specialists in various applied fields and fundamental scientists, and also cause public resonance, the number of works devoted to these topics is growing explosively.

## Abstract (ru)

Быстро накапливающийся массив геномных данных – секвенированных геномов сельскохозяйственных растений – позволил получить обширные результаты по геномному прогнозированию и выявлению ассоциаций однонуклеотидных полиморфизмов с фенотипическими признаками. Во многих случаях для обнаружения новых связей фенотипов с генотипами предпочтительно использовать методы машинного обучения, глубокого обучения и искусственного интеллекта, в особенности объяснимого, способные распознавать сложные закономерности. Вручную было отобрано 80 источников, при этом ограничения по дате выхода не ставилось, основной интерес представляла оригинальность предлагаемого подхода или модификации для применения в задаче геномного прогнозирования. В статье рассмотрены модели для геномного прогнозирования, сверточные нейронные сети, объяснимый искусственный интеллект и большие языковые модели. Уделено внимание подходам к дополнению данных, переносу знаний, методам снижения размерности и гибридным методам. Приведен пример современного способа кодирования больших геномных данных в искусственные изображения, преимуществом которых являются наглядная визуализация и возможность использования известных моделей для извлечения признаков. Исследования в области модель но-специфичных и модельно-независимых методов интерпретации решения моделей представлены тремя основными категориями: зондирование, возмущение и суррогатная модель. В рассмотренных примерах отражены основные современные тренды в изучаемой области. Отмечены растущая роль больших языковых моделей, в том числе основанных на трансформерах, для обработки генетического кода, а также разрабатываемые методы аугментации данных. Дополнительным преимуществом применения языковой модели может стать возможность формулировать запросы на близком к естественному языке и получать ответы за относительно короткое время. Среди гибридных подходов выделена перспективность сочетания моделей машинного обучения и моделей развития растений на основе биофизических и биохимических процессов. Поскольку методы машинного обучения и искусственного интеллекта находятся в фокусе внимания как специалистов в различных прикладных областях, так и фундаментальных ученых, а кроме того, вызывают общественный резонанс, количество посвященных этим темам работ имеет взрывной рост.

## Introduction

List of abbreviations SNP – single nucleotide polymorphism GP – genomic prediction GBLUP – genomic best linear unbiased predictor ML – machine learning RRBLUP – ridge regression with best linear unbiased predictor CNN – convolutional neural network AIO – artificial image object PCA – principal component analysis XAI – Explainable Artificial Intelligence DT – decision trees RF – random forest LLM – large language model GPT – generative pretrained transformer

To this day, a tremendous amount of genomic data has been accumulated and it continues to grow rapidly. These data include the sequenced genomes of agricultural plants such as chickpea, vigna, soybean, wheat, rye, flax etc. (Bragina et al., 2019; Ichihara et al., 2023; Chamorro-Padial et al., 2024; Tang et al., 2024). Many annotations have been obtained, classical methods of genomic prediction and genome-wide association studies have been successfully applied to these data, and SNPs associated with different important phenotypes have been identified (Hayes, 2013).

Many phenotypic traits that selection programs are targeted to are correlated and thus require use of multi-trait models in order to obtain statistically significant predictions. Machine learning methods are suitable for such a class of problems as well as deep learning models and artificial intelligence, explainable AI in particular, which are able to recognize complex patterns in the given data and generalize extracted knowledge

**Fig. 1.**
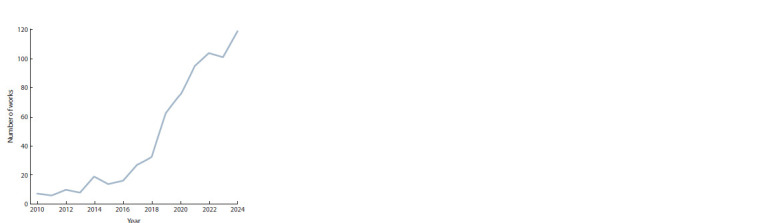
The growth of the number of works in PubMed

The papers for the current review were selected based on the originality of the proposed approach or modification for application to the solution of the genomic prediction problem. The search was performed in PubMed (https:// pubmed.ncbi.nlm.nih.gov/, accessed on November 7, 2024) using terms “plants genomic prediction machine learning” and dates from the beginning of the year 2010 to the end of the year 2024, which showed exponential growth of the number of manuscripts per year with a small decrease in the growth rate after the year 2021 (Fig. 1).

**Fig. 2.**
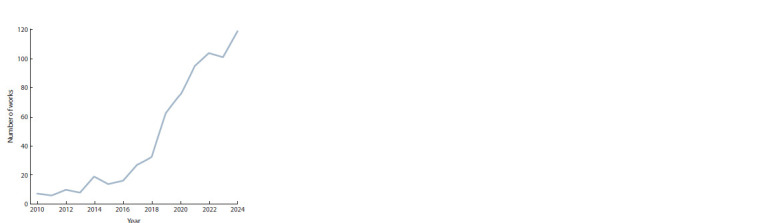
The distribution of the selected works over the years.

Eighty sources were selected manually without restrictions on the publication date. The oldest manuscript was published in the year 1988, the majority of works (60 %) were published after the year 2020, and 20 % of the reviewed papers belong to the last two years (Fig. 2).

## Genomic prediction

Genomic prediction (GP) aims to predict the phenotype of an organism given single nucleotide polymorphism (SNP) data (Meuwissen et al., 2001). The wide range of genomic prediction methods can be divided into two groups: linear and nonparametric. Linear methods such as BLUP work well for additive traits. They model the phenotype as a function of the contributions of different factors such as individual markers, weather parameters, field conditions, etc. On the other hand, nonparametric machine learning methods such as support vector machines, random forests, and gradient boosting can model nonlinear traits, providing great flexibility to accommodate complex genotypephenotype associations (Montesinos-López et al., 2021).

Genomic prediction tools based on statistical methods such as genomic best linear unbiased prediction (GBLUP) are widely used in crop breeding. However, these tools are not designed to account for nonlinear relationships in high-dimensional datasets or to handle high-dimensional datasets such as drone images. Machine learning (ML) algorithms have the potential to surpass the prediction accuracy of current tools used to predict phenotypic traits from genomic data due to their ability to autonomously extract features and represent their relationships at multiple levels of abstraction (Danilevicz et al., 2022).

The accuracy of prediction depends on the quality and pre-processing of phenotypic data, the platform used to obtain genomic information, the population breeding scheme, the internal genetic architecture of the trait, the genetic structure of the population, how genotype-environment interactions are treated, and the prediction method (de Los Campos et al., 2013).

It was reported in (Sandhu et al., 2021) that deep learning models outperformed traditional ridge regression with best linear unbiased prediction (RRBLUP) and Bayesian models under all forecasting scenarios. Machine learning methods were used to increase the statistical power of the models. To apply multi-stage machine learning, a new BioM2 package (Zhang S. et al., 2024) was proposed for the statistical computing system R, which has the ability to apply stratification and aggregation of multivariate data based on biological information to improve the training efficiency of models. In this case, stratification allows one to build subsets of data, for example, training and test samples, by controlling the ratio of the number of objects from different groups, for example, SNPs in genes involved in different processes.

At the same time, aggregation of multivariate data makes it possible to use simpler and more easily interpretable models that can be refined during multi-stage training. An innovative computational framework, PlantMine, which combines feature selection and machine learning methods to efficiently identify key SNPs, was proposed in (Tong et al., 2024), taking critical factors for trait improvement in rice as an example. Various data mining algorithms were applied to the 3,000 Rice Genomes Project dataset. The results highlighted the effectiveness of combining feature selection with machine learning to accurately identify key SNPs, offering a promising avenue to accelerate the breeding of new plant varieties with improved yield and stress tolerance. The overall model performance depended more on the prediction algorithm than the predictor selection method. Among all the models, decision tree-based machine learning methods (random forests and gradient boosting) performed the best, while classical Bayesian methods were prone to overfitting (Sirsat et al., 2022).

## Convolutional neural networks and artificial image objects

Among machine learning methods, convolutional neural networks (CNNs) provide the best ability to discover hidden patterns or features from data and are best suited for image analysis (Pook et al., 2020; Montesinos-López et al., 2021). Artificial image objects (AIO) are a new concept for genomic data representation that can be used to encode large genomic data by treating individual genetic variants as pixels (Galli et al., 2022). The advantages of AIOs include convenient, simple visualization, compactness, and the ability to apply a wide range of methods developed for image analysis and classification (Chen X. et al., 2021b), in particular CNNs (Chen X. et al., 2021a). Therefore, AIOs can be used by CNNs for regression and classification tasks (Bavykina et al., 2022).

The algorithm for optimization of data packing in AIO was proposed in (Bazgir et al., 2020). The DeepFeature package proposed in (Sharma et al., 2019, 2021) was developed to transform large-scale experimental data, such as genomic or transcriptomic data, into a form optimal for training a CNN model. The input vector is transformed into a matrix using t-SNE, kernel PCA, PHATE, or UMAP, and the smallest rectangle containing all elements is found using the convex hull algorithm. A rotation is performed to flatten the image, converting Cartesian coordinates into pixel indices.

The application of CNN to AIO processing enables the calculation and visualization of the influence of various factors on the final solution of the model. The work of (Liu et al., 2019) was considered to be the first study to apply the saliency map to identify the most important predictors in soybean. In this study, gaps in the data were treated as a new genotype; as a result, each SNP was encoded with four binary values. The significance value of each genotype was calculated as the maximum absolute value of the gradients among these four encoding channels, and the population median was used as a measure of the contribution of the SNP.

The ResNet architecture, widely used in deep learning methods, was adapted for use in genomic selection models in (Xie et al., 2024). Since each locus makes a different contribution to the final phenotype, successive convolutions are more suitable for the genomic selection model than layer pooling. Thus, a deep learning algorithm, ResGS, was proposed that significantly alleviates the problem of degradation, i. e., the decrease in performance with increasing model depth, which can improve the prediction accuracy compared to traditional methods (Wu H. et al., 2024).

Recently, more and more attention has been paid to the internal mechanisms of convolutional neural networks and the reasons why the network makes certain decisions (Wang et al., 2020). Several methods have been proposed, including data permutation and backpropagation approaches (Zhang X., Gao, 2020), gradient-based algorithms (Selvaraju et al., 2020), and class activation maps (Wang et al., 2020). A saliency map represents the spatial regions associated with a particular class in a given image (Simonyan et al., 2014). Class activation maps provide a visual explanation for a single input image (Chattopadhay et al., 2018; Selvaraju et al., 2020), but are sensitive to the model architecture. Gradient-weighted class activation mapping (Grad-CAM) uses the gradients of any target concept fed to the final convolutional layer to create a coarse localization map, which highlights important regions in the image for class prediction (Selvaraju et al., 2017).

Score-CAM, unlike previous class activation mappingbased approaches, removes the dependence on gradients by deriving the weights of each activation map by directly computing the network for instances of the target class, with the final output being a linear combination of the weights and activation maps (Wang et al., 2020). Grad-CAM++ (Chattopadhay et al., 2018), a modification of Grad-CAM (Selvaraju et al., 2020), generalizes CAM to models without global pooling layers. LayerCAM (Jiang et al., 2021) can generate robust class activation maps from a combination of class activation maps from different CNN layers.

## Explainable Artificial Intelligence

Explainable Artificial Intelligence (XAI) aims to overcome the black box problem and provide insight into how AI systems make decisions. Interpretable ML models can explain how they make predictions and identify the factors that influence their results. However, most modern interpretable ML methods were developed for domains such as computer vision, making direct application to bioinformatics problems difficult without customization and domain adaptation.

An interpretable ML model can identify the factors that influence its output (e. g. statistically significant features) and explain the interactions between them (Molnar, 2022). Depending on the level of abstraction, methods can be divided into local and global interpretability methods. While local methods focus on interpreting individual predictions, global ones try to explain the behavior of the entire model in the form of diagrams or lists. Various variants of modelspecific and model-independent interpretable ML approaches have been developed, on which an XAI system can be built to improve its local and global interpretability (Wachter et al., 2018), but these methods are most often used to improve visualization (Weber et al., 2023). Linear models, decision trees (DTs), and rule-based systems are less complex and inherently interpretable. However, they are less accurate compared to tree-based ensembles such as random forests (RF) and deep neural networks, resulting in a trade-off between accuracy and interpretability.

Many specific and model-independent interpretable ML methods have been developed (Azodi et al., 2020). All these methods can be divided into three main categories: probing, perturbation, and surrogate models. Examples of probing methods are gradient-based methods such as gradient- weighted class activation mapping (Grad-CAM++) and layered relevance propagation (LRP) (Guidotti et al., 2018). A widely used perturbation-based method is Shapley additive explanations (SHAP). SHAP is based on coalition game theory, i. e., on the average marginal contribution of a feature and the way the payoffs are distributed among its players (Cubitt, 1991).

Since interpretability comes at the cost of a trade-off between accuracy and complexity, studies have proposed training a simple interpretable model to imitate a complex model (Molnar, 2022). A surrogate or simple proxy model is a model interpretation strategy that involves training an initially interpretable model by approximating local black box predictions (Stiglic et al., 2020; Molnar, 2022).

The majority of surrogate model building tools were developed with the aim of improving the interpretability and explainability of black-box ML models covering common problems in computer vision, text mining or structured data, and were based on well-known interpretable ML methods such as LIME (Ribeiro et al., 2016), Model Understanding through Subspace Explanations (MUSE) (Lakkaraju et al., 2019), SHAP (Lundberg, Lee, 2017) (and its variants such as SHAP kernel and SHAP tree), Partial Dependency Graph (PDP), Individual Conditional Expectation (ICE), Permutation Feature Importance (PFI) and Counterfactual Explanations (CE) (Wachter et al., 2018).

## Large language models

Recently, the use of large language models (LLM) has become widespread in various fields of science, including decoding genetic text to predict the manifestation of useful traits in plants. LLMs, such as GPT-4, have conquered the world, demonstrating amazing capabilities in natural language proficiency, which immediately prompted researchers to adapt LLMs to a different type of language – the genome, in order to solve complex problems based on large volumes of accumulated data. The success of LLMs is largely due to the use of transformer-based attention units in the architecture. The use of such architectural solutions allowed the well-known AlphaFold2 neural network (Google DeepMind, 2021) to predict three-dimensional protein structures with unprecedented accuracy. Alpha- Fold3 (2024), according to the developers, for the first time surpasses physical methods in its prediction of the 3D structure of proteins, as well as the interactions of proteins with each other and with other substances. Profluent’s LLM has made it possible to create an artificial protein for genome editing that is comparable in efficiency to the natural one, but has much greater specificity.

The broad implementation of the results of these achievements in production requires a deep understanding of the underlying mechanisms, taking into account complex interactions, accelerating the search for answers to questions arising in practice. In particular, there is a need to shift from identification of SNPs associated with a trait to identification of genes that affect the trait with a greater degree of reliability. In addition, it is necessary to take into account the gene-gene interactions, and to consider not only one trait, but also pairs of related traits. The solution to the described problem is impossible without involving the latest accomplishments in computer science, such as artificial intelligence based on large language models. An additional advantage of using such an approach is the ability to formulate queries in a language close to a natural one and receive answers in a relatively short time.

Research in this area has increased significantly in recent years. For example, a review (Consens et al., 2023) on the application of transformer-like models to genetic data included more than 100 recent papers and noted rapid development in the field. The use of large language models based not only on transformers, but also using the so-called Hyena layer (Poli et al., 2023) to process genomic data was also noted (Nguyen et al., 2023). One interesting approach is the possibility of pre-training such models on genome sequences without using phenotypes.

Currently, the maximum input sequence length among publicly available DNA transformer-based LLMs is limited to only 3 × 104 nucleotides for the GENA-LM architecture. To mitigate this limitation, the performance of a modified recurrent memory transformer (RMT) architecture in the GENA-LM model was studied in (Kuratov et al., 2024) for multiple genomic analysis tasks requiring processing of long DNA sequences. The results obtained in (Kuratov et al., 2024) showed that augmenting GENA-LM with RMT leads to a significant performance improvement.

A new method based on a transformer-like neural network to predict the severity of fusarium and the associated accumulation of the dangerous mycotoxin deoxynivalenol was proposed (Jubair et al., 2021) that used genomic and phenotypic data on the barley. The work showed the superiority of frequency coding of markers and mentioned the high memory requirements of the model when using a large number of markers, which could be reduced using selection by the information criterion.

In the paper (Wu C. et al., 2023), a genomic selection model based on a deep neural network using transformers, convolutional layers, and an additional information module was proposed. The model architecture used encoding of marker positions with trigonometric functions, fast Fourier transform, Gaussian linear activation function (GELU), and included blocks of convolutional network, transformer, and regressor. The model was applied to five datasets, where it outperformed the four methods used for comparison.

An important source of the phenotype prediction accuracy reduction in models based on genomic data is the lack of gene-gene interactions consideration. The work (Cui et al., 2022) proposed an approach for identifying interactions between genes and taking them into account in a deep learning model for phenotype prediction. A layer representing genes as hidden nodes of a sparse network was added to the deep neural network architecture. Importantly, the Shapley values for hidden nodes of the gene layer were used to determine the influence of interactions on the model solution.

## Data augmentation

Training large language models requires a large amount of data because there is a large number of unknown parameters. The papers (Jubair et al., 2021) and (Wu C. et al., 2023) consider transformer-like neural network-based models for genomic prediction. In the paper (Jubair et al., 2021), GPTransformer contains two Transformer encoding blocks, uses two nodes for each attention layer, and each Transformer block contains 256 hidden neurons. The output is a vector, which is the input of a feedforward network, which contains one output neuron. The mean squared error (MSE) loss function is used. A dataset of 400 genotypes phenotyped in 3 geographic areas and 2 years, i. e. 2,400 records, was used for training and analysis, and the Pearson correlation coefficient between the model prediction and the data was 0.6, which allowed obtaining significant results

The GPformer model (Wu C. et al., 2023), based on the transformer-like neural network for predicting phenotype from genotype, was separately trained and tested on the Soybean999, Maize282, Rice469, Wheat599 and Wheat2403 datasets, which have 999, 282, 469, 599 and 2,403 records, respectively. The resulting Pearson correlation coefficient was 0.4–0.8 for different variants.

An additional tool, as in the case of deep learning models for image processing, can be data augmentation, which has recently been studied for deep learning models in the field of bioinformatics. For example, a new approach to augmentation of biological sequence data was proposed in (Ji et al., 2024), in which the chromosome order is changed. This method of generating additional data can be used for training, because the models cannot use the chromosome number as a predictor. In the work (Montesinos-López et al., 2024) a blending method was considered, which offers a domain-independent approach to augmentation based on the assumption that a linear combination of feature vectors should approximately correspond to a linear combination of their corresponding target values. In (Vilov, Heinig, 2022), data augmentation was successfully used to train a classifier of genomic variants. The approaches based on a generative network (GAN) and a Boltzmann machine (RBM) for compiling synthetic genomes were presented in (Yelmen et al., 2021). In the mentioned works, the authors managed to improve the accuracy and generalization ability of the models, so data augmentation can be used to expand the existing dataset for training the LLM.

A new method was proposed to predict the classification of enhancers into strong and weak using data augmentation and a convolutional neural network ES-ARCNN (Zhang T.-H. et al., 2021). Two data augmentation techniques, such as reverse augmentation and shifting, were used to train ES-ARCNN for previously identified enhancers.

## Transfer learning

Transfer learning enables the creation of effective models for a target domain using knowledge from a different but related source domain. In medical research, knowledge transfer can significantly improve the accuracy of disease prediction for data-poor populations with imbalanced data (Gao, Cui, 2022). This approach also has great potential to improve the prediction of complex phenotypic traits, such as plant yield, although it does not work in all cases (Kovalev et al., 2018). Transfer learning is widely used to extract features from images with the models pre-trained on general-purpose datasets and then fine-tuned on a relatively limited, specialized dataset (Kirchler et al., 2022).

To facilitate the application of the Transfer learning approach to phenotype-to-genotype prediction models, an efficient implementation of TrG2P was proposed in (Li et al., 2024). In the developed framework, firstly, convolutional neural networks were trained using genomic data and phenotypic traits with simpler dependencies than a complex target trait, such as yield. Then, the parameters of the convolutional layers of these pre-trained models were transferred to the target trait prediction task, and the fully connected layers were retrained, thus leading to improved prediction accuracy of the resulting model (Li et al., 2024).

## Dimensionality reduction methods

The explosive growth of available amounts of data not only brings unprecedented progress in bioinformatics and opportunities to perform predictive modeling (Han, Liu, 2022), but also poses challenges to existing AI methods and tools, such as data heterogeneity, high dimensionality, and volume (Karim et al., 2021). Principal component analysis (PCA) and isometric feature mapping (Isomap) are widely used as dimensionality reduction methods (Fournier, Aloise, 2019). However, the representations obtained by these methods often lose essential properties (Aggarwal, Reddy, 2014), making them less effective against a well-known phenomenon called the curse of dimensionality, especially for high-dimensional datasets (Fournier, Aloise, 2019).

## Hybrid methods

With increasing computing power, existing machine learning approaches are frequently combined into complex hybrid models. For example, (Chen C. et al., 2024) considered algorithms that first use BayesR/GWAS to identify a subset of 1,000 markers with moderate to large marginal additive effects, and then use attention networks to make predictions based on these effects and their interactions. Hybrid methods with attention networks yielded the lowest variance in prediction accuracy across all validation datasets and the lowest root mean square error, the criteria usually applied in practical breeding programs. In (Ramzan et al., 2020), a two-step procedure was proposed to solve the problem of detecting a large number of loci with small effects on the phenotype. In the first step, the Wald test statistics values are approximated by cubic splines, and genomic regions with spline’s extrema that are higher than expected are considered as quantitative trait loci (QTLs). SNPs in these QTLs are then ranked by their association with the phenotype using a random forest approach. In the work (Nascimento et al., 2024), a Stacking Ensemble Learning (SEL) model was proposed, which combines several models that can potentially predict important traits more accurately than individual ones; the model was applied to the example of coffee breeding in Coffea arabica.

A recently proposed direction of research is the combination of machine learning models and crop growth models based on biophysical and biochemical processes (CGM). It has been suggested that such an approach can improve the predictions of integrative traits by decomposing them into simpler intermediate traits with better heritability (Larue et al., 2024). In the study, the combined CGMGP model outperformed the genomic selection models without CGM integration in the predictive ability, regardless of the regression method used. CGM simulates nonlinear (causal) plant responses to the environment through model parameters (representing genotypic sensitivity to these responses, G×E). Thus, calibrated CGMs for a genotype can be useful for predicting its performance under unknown conditions; on the other hand, it is impossible to predict the performance of unknown genotypes (Larue et al., 2019).

## Conclusion

The great variety of machine learning and artificial intelligence methods finds applications in the field of bioinformatics of agricultural plants for such problems as genomic prediction of important phenotypic traits. ML and AI attract close attention of researchers and practitioners from different areas as well as cause resonance in the public, and consequently the number of published manuscripts grows explosively.

The main contemporary trends in the field of ML and AI for GP were included in the review. The examples of the application of common machine learning models and their variants modified for bioinformatics tasks were considered. These examples illustrated the usage of the ML and AI methods alone and in combination with dimensionality reduction and feature selection approaches, the construction of explainable AI solutions and developing hybrid methods. The increasing role of large language models deserves a separate mention, including those based on transformers, and the associated data augmentation methods needed to train models with a huge number of parameters. Transfer learning methods can be used to mitigate the problem of insufficient or imbalanced data.

An important aspect of ML and AI success is data representation, for example, the artificial image objects described in the review make it possible to utilize the powerful and highly efficient apparatus of convolutional neural networks for extraction of characteristic patterns from the data. Such an approach also allows ranking the importance of predictors based on attention maps.

With the rise of the Internet of things, the spread of mobile devices and autonomous robots, a new trend of edge computing started to evolve, seeking solutions to the compactization of models and optimization of algorithms for resource-limited devices. This topic deserves a separate review and was not considered in the current work.

## Conflict of interest

The authors declare no conflict of interest.
